# Genetic Variants Influencing Individual Vitamin D Status

**DOI:** 10.3390/nu17162673

**Published:** 2025-08-19

**Authors:** Niel A. Karrow, Spencer E. Leuschner, Umesh K. Shandilya, Bonnie A. Mallard, Lauraine Wagter-Lesperance, Byram W. Bridle

**Affiliations:** 1 Department of Animal Biosciences, University of Guelph, Guelph, ON N1G 2W1, Canada; sleuschn@uoguelph.ca (S.E.L.); ushand@uoguelph.ca (U.K.S.); 2 Immunoceutica Inc., Cambridge, ON N1T 1N6, Canada; bmallard@ovc.uoguelph.ca (B.A.M.); lwagterl@uoguelph.ca (L.W.-L.); bbridle@uoguelph.ca (B.W.B.); 3 Department of Pathobiology, University of Guelph, Guelph, ON N1G 2W1, Canada

**Keywords:** vitamin D metabolism, genetic polymorphisms, CYP450 enzymes, genomic risk score

## Abstract

Vitamin D (VD) plays a critical role in human health, with deficiencies linked to a range of adverse outcomes, including compromised immune function and increased disease risk. While environmental factors such as sunlight exposure and diet influence circulating VD levels, genetic variation is a significant and underappreciated contributor to interindividual differences in serum 25-hydroxyvitamin D [25(OH)D] concentrations. This review provides a comprehensive summary of genetic variants in key genes involved in VD synthesis (e.g., *DHCR7*, *cyp2r1*, *cyp27b1*), transport (*GC*), and metabolism (*cyp24a1*, *cyp3a4*), as well as in cholesterol transport proteins (*SCARB1*, *CD36*, *NPC1L1*). We examine how single-nucleotide polymorphisms (SNPs) and rare mutations in these genes affect enzyme activity, VD bioavailability, and overall 25(OH)D status. Importantly, we highlight evidence supporting gene-by-environment interactions and population-specific allele frequencies that further shape individual VD responses. In the context of clinical nutrition and precision health, these findings support the development of genomic risk scores (GRSs) to identify individuals at risk for deficiency or toxicity and guide personalized VD supplementation strategies. Regular monitoring of serum 25(OH)D alongside genetic screening may improve clinical outcomes by helping to achieve optimal VD immunosufficiency while minimizing the risk of adverse effects.

## 1. Introduction

Humans are efficient producers of vitamin D (VD) under ideal conditions, which requires direct exposure to ultraviolet B (UVB) radiation in the form of relatively intense sunlight. At northern and southern latitudes that are distal to the equator, however, the amount of direct sunlight exposure and skin temperature are insufficient to facilitate adequate production of cholecalciferol (VD_3_) for many months of the year, and without dietary sources, this can lead to VD deficiency and negative health outcomes such as compromised immunological functions. A recent study highlighted the critical impact of season on circulating VD concentrations [1], which is relevant to anyone living relatively far from the equator. In this study, VD status of Slovenian adults (18–64 years of age) and the elderly (65–74 years of age) was assessed by analysis of serum calcidiol (calcifediol; 25(OH)D) concentration. Since calcidiol is the major circulating VD metabolite, is the most stable and has the longest half-life [2], measured in terms of ≅15 days versus 10–20 h for calcitriol (1α,25(OH)_2_D_3_), the bioactive form of VD [3] and its concentrations are most commonly monitored in individuals. Based on their classification of VD deficiency (<12 ng/mL) and insufficiency (<20 ng/mL), Hribar et al. reported that during the winter months (November–April), adults and the elderly were VD deficient (40.8% and 34.6%) and insufficient (81.6% and 78.8%), respectively, which prompted them to recommend policy interventions to mitigate what they deemed to be a public health risk [1].

VD signaling, mediated by the VD receptor (VDR), is responsible for modulating the expression of thousands of genes, including many that regulate the immune system [4]. A circulating calcidiol concentration above 50 ng/mL has been proposed as the minimum concentration required for its uptake into peripheral target cells, such as leukocytes and intracellular calcitriol synthesis [5,6,7,8]. Several studies have suggested that maintaining serum calcidiol concentrations in the range of approximately 60 ng/mL may be associated with reduced risk of various health conditions, including certain cancers and pre-eclampsia [4,9,10,11,12,13]. However, the definition of “optimal” VD status remains a matter of active debate, and proposed thresholds vary among expert groups and studies. At the other end of the spectrum, the Endocrine Society previously proposed a cut-off of 150 ng/mL of calcidiol in blood, above which they postulate a person may be at potential risk of toxicity in the form of hypercalcemia [14].

In addition to seasonal and dietary factors influencing VD concentrations, genetics also contribute to individual variation in circulating VD, which is predetermined by polymorphisms and mutations within genes coding proteins regulating VD synthesis, transport, and metabolism. As such, this paper will provide a state-of-the-art review of the genetic mechanisms that contribute to individual variation in circulating VD concentrations, with the intent to help identify individuals at risk of VD deficiency or potential VD toxicity.

## 2. Synthesis, Regulation, and Metabolism of Vitamin D

VD can be obtained from three different sources, including endogenous synthesis in skin during exposure to sunlight, diet, and dietary supplements [15]. VD is typically consumed as cholecalciferol from foods of animal origin and dietary supplements, and ergocalciferol (VD_2_) from foods of plant origin, which can also be supplemented [15]. The most common dietary sources of VD are oily fish and fish products such as salmon, mackerel, and cod liver oil, as well as foods fortified with cholecalciferol, such as fortified milk, cheeses, and yogurts [15].

**Figure 1:**
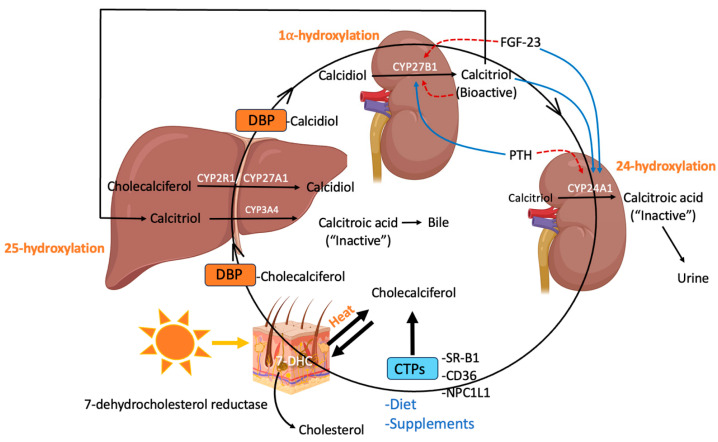
Synthesis, transport, and metabolism of vitamin D throughout the body (created using BioRender): 7-dehydrocholesterol (7-DHC), cholesterol-binding proteins (CTPs), scavenger receptor class B type 1 (SR-BI), Niemann-Pick C1-Like 1 (NPC1L1), vitamin D binding protein (DBP), fibroblast growth factor (FGF)-23 (FGF-23), parathyroid hormone (PTH).

Humans can efficiently produce endogenous VD under ideal conditions. When the skin is exposed to UVB radiation (290–315 nm), 7-dehydrocholesterol (7-DHC) is converted to the pro-hormone pre-VD_3_, which undergoes thermal isomerization to produce cholecalciferol, optimally at 37 °C (Tsiaras and Weinstock 2011 [16]). This reaction is reversible, allowing pre-VD_3_ and cholecalciferol to exist in equilibrium, but the equilibrium constant (K = 11.44 at 37 °C) favors cholecalciferol synthesis [17,18]. Further irradiation with UVB converts pre-VD_3_ to isomers of lumisterol (i.e., L3) and tachysterol (i.e., T3) [19], and cholecalciferol to inactive suprasterols [20]. This mechanism, in part, allows for the regulation of VD produced in the skin, and avoidance of toxicity [20]. The conversion of pre-VD_3_ to L3 is also reversible, allowing for the replenishment of cholecalciferol when its concentrations diminish as UV irradiation ceases [20]. Alternatively, 7-DHC can be converted to cholesterol by the enzyme 7-dehydrocholesterol reductase (DHCR7) (Figure 1) [17]. By controlling the availability of 7-DHC, DHCR7 serves as a regulator of cholesterol homeostasis and is a key regulatory switch between cholesterol and VD synthesis. DHCR7 converts 7-DHC to cholesterol, but when exposed to UVB radiation, 7-DHC is converted to cholecalciferol in the skin.

In vitro studies have investigated regulation of DHCR7, and its link to VD synthesis. Treatment of human keratinocyte (HaCaT) cells with cholesterol, for example, reduced DHCR7 activity [21]. This resulted in an accumulation of 7-DHC and increased cholecalciferol concentrations due to a decrease in the cholesterol synthesis pathway involving 7-DHC and DHCR7 [21]. Ultraviolet irradiation of HaCaT cells also reduced DHCR7 mRNAs and proteins and increased calcitriol synthesis [22]. It has also been shown that UV-irradiated human skin has reduced DHCR7 mRNAs [22]. Additionally, DHCR7 activity was decreased in human epidermal keratinocytes (HEKa) when treated with cholecalciferol [23], with the suggestion that extracellular cholecalciferol reduces DHCR7 activity by binding to the G protein-coupled receptor Smoothened (Smo) and modulating the Hedgehog signaling pathway. However, definitive in vivo evidence has yet to be elucidated. Taken together, cholesterol, UV irradiation, and cholecalciferol all contribute to regulating DHCR7 and influence VD synthesis in the skin.

At latitudes distal to the equator, the intensity of sunlight on exposed skin is insufficient to facilitate adequate production of cholecalciferol in the epidermis for many months of the year, which is worsened by cool air and clothing that conceals the skin, and is most critical for those with darker skin tones having more pigment-producing melanocytes. It is hypothesized that skin tone lightened over time after human migrations occurred out of Africa to help improve VD production under suboptimal environmental and restricted dietary conditions [24,25]. Without sufficient dietary sources, exposure to only low intensity sunlight can lead to VD deficiency with negative health outcomes [26,27].

As VD is lipid soluble, the absorption of dietary and supplemented VD is enhanced by the presence of lipids. Absorption requires the release of gastric lipase to hydrolyse esterified VD [28]. Subsequently, in the small intestine, bile acids and pancreatic lipases promote the formation of mixed micelles that solubilize free VD and support its transport across intestinal enterocytes [28]. Murine studies suggest that the main site of VD absorption is the jejunum [29,30]. Although passive diffusion is thought to be the mechanism of transport for VD across enterocytes at high concentrations, uptake of VD at low concentrations is mediated by cholesterol-transport proteins (CTPs) on intestinal epithelial cells (Figure 1), including Scavenger Receptor Class B type 1 (SR-BI), CD36 and Niemann-Pick C1-Like 1 (NPC1L1) [30]. Once absorbed, VD is packaged into chylomicrons and transported across the intestinal epithelium to the portal vein or indirectly to the blood via the lymphatic system, where it then circulates throughout the body [28]. In circulation, cholecalciferol binds to transport proteins that carry VD to the liver (Figure 1). Due to its high binding affinity, VD binding protein (DBP) is responsible for transporting ~85% of VD, while albumin transports ~15% leaving only ~0.4% free VD in the circulation [31].

Enzymes in the cytochrome P450 (CYP) superfamily are responsible for generating various bioactive and inactive VD metabolites. In the liver, cholecalciferol is hydroxylated by CYP2R1 and CYP27A1 to form calcidiol, which is transported to the kidneys, primarily by DBP, for further hydroxylation (Figure 1) [2]. In the kidneys, calcidiol is hydroxylated to calcitriol by CYP27B1 [2], which mediates its physiological effects by binding to the VDR that regulates target gene expression using complex genomic and nongenomic mechanisms [32,33]; other VD metabolites are also able to bind and activate the VDR with varying potencies [34]. Membrane receptors, such as protein disulfide-isomerase A3 (PDIA3), have also been implicated in the rapid non-genomic responses to calcitriol including rapid cellular calcium influx [33]. CYP11A1 hydroxylates VD_3_ to produce numerous bioactive VD metabolites in mammals, such as 20(OH)D_3_ that can be further hydroxylated by CYP27B1 to 1,20(OH)_2_D_3_ [19,35]. Calcidiol and calcitriol can also be metabolized by CYP24A1, primarily in the kidneys, to produce 24,25(OH)_2_D_3_ and 1,24,25(OH)_3_D_3_, respectively. *Cyp24a1* gene expression has also been detected in the skin, intestine, bone, and cells of the immune system [36,37]. After further hydroxylation, 1,25(OH)2D-26,23-lactone or calcitroic acid are produced and excreted from the body (Figure 1) [38]. CYP3A4 is also expressed in the liver and small intestine and catalyzes the metabolism of calcidiol and calcitriol to the inactive VD metabolites 4,25(OH)_2_D_3_, 1,23,25(OH)_3_D_3_ and 1,24,25(OH)_3_D_3_, respectively, with subsequent excretion from the body [39,40,41].

Excretion of VD metabolites first requires conjugation reactions such as glucuronidation and acetylation [42]. Conjugation of VD metabolites in the liver leads to intestinal secretion via bile, which is the predominant route of excretion for metabolites such as calcitroic acid, while conjugation in the kidney leads to urinary excretion [42]. Although metabolites of dietary ergocalciferol are carried throughout the body and metabolized in similar ways to cholecalciferol, ergocalciferol metabolites have a weaker affinity for DBP and the VDR [43], and this is a reason why cholecalciferol supplementation is more effective at raising serum calcidiol concentrations [44].

The CYP enzymes responsible for VD metabolism are tightly regulated. *Cyp2r1* gene expression is generally thought to be unregulated, whereas *cyp27a1* gene expression is regulated by hepatocyte nuclear factor 4α in the liver and the pregnane X receptor (PXR) in other tissues [2,45,46]. Constitutive expression of the *cyp27b1* and *cyp24a1* genes in extrarenal tissues is typically restricted to low levels. However, *cyp24a1* can be induced by calcitriol in non-renal cells [37,47]. In the kidneys, *cyp27b1* and *cyp24a1* genes are tightly regulated to control the concentration of circulating calcitriol, which ensures VD and calcium/phosphate homeostasis are maintained [37,47]. Calcitriol and fibroblast growth factor (FGF)-23 are both responsible for downregulating *cyp27b1* and upregulating *cyp24a1* expression (Figure 1). Upregulation of *cyp24a1* by calcitriol is mediated via a pair of VD response elements (VDREs) within its promoter region [48]. In contrast, parathyroid hormone (PTH) is responsible for upregulating *cyp27b1* and downregulating *cyp24a1* [37,47]. The *cyp3a4* gene is highly expressed in the liver and small intestine, and is under the influence of several transcription factors, including the VDR. *Cyp3a4* expression is upregulated via the VDR-mediated pathway and is affected by seasonal variation that correlates with VD levels [41].

## 3. Genetic Variants Affecting Vitamin D Status

### 3.1. Candidate Gene and Genome-Wide Association Studies

Many candidate gene (CGAS) and genome wide (GWAS) association studies have been carried out to identify polymorphisms in the *VDR* associated with skeletal and non-skeletal health. However, as pointed out by Jolliffe et al., information regarding associations with genes involved in the synthesis, transport, and metabolism of VD are lacking [49]. In their review, Jolliffe et al. identified 44 studies reporting associations between single nucleotide polymorphisms (SNPs) in *DHCR7*, *GC* which codes for DBP, *cyp2r1*, *cyp24a1* and *cyp27b1*, and circulating VD concentrations [49]. Another recent review by Hyppönen et al. of 29 GWAS reported loci harboring *DHCR7*, *GC*, and *cyp2r1* that were consistently associated with calcidiol concentrations in blood across European and non-European groups, and across age groups [50]. These authors also discussed the heritability of calcidiol concentrations in blood, which varies greatly depending on study design. They reported that for twin- and family-based studies, heritability estimates range from 0.28 to 0.80. In contrast, heritability estimates from a large UK Biobank GWAS involving unrelated white Europeans only ranged from 0.13 to 0.16 [50]. Importantly, the authors also commented on gene-by-environment interactions that can affect GWAS. This included seasonal variation affecting calcidiol concentrations, which needs to be taken into consideration during study design, since genes affecting skin tone could differentially impact seasonal calcidiol levels. It is important to note that GWAS and CGAS findings are not always concordant, partly due to differences in study design, statistical power, population ancestry, and environmental exposures. Likewise, heritability estimates from family- and twin-based studies often exceed those from large GWAS of unrelated individuals, reflecting methodological differences rather than true biological discrepancies. Reconciling these findings requires replication across diverse cohorts and the integration of multiple study types to provide a more comprehensive picture of the genetic contributions to VD status.

It is important to distinguish between genetic correlations observed in association studies and proven causal mechanisms. While GWAS and CGAS can identify loci statistically associated with circulating 25(OH)D, such associations may reflect linkage disequilibrium with the causal variant or indirect effects mediated through related biological pathways. Establishing causality requires additional evidence from functional genomics, Mendelian randomization, or experimental validation to confirm that a given SNP directly influences VD metabolism.

The following sections will discuss variants in individual genes and their potential mechanisms affecting VD concentrations in blood. To the best of our ability, reference sequences (rs) have been included for each genetic variant, and up-to-date information has been provided for the gene location of the variant based on current data provided in the NCBI dbSNP database (GRCh38p.14 (NC_000007.14); Table 1).

### 3.2. 7-Dehydrocholesterol Reductase (DHCR7)

*DHCR7* is located on human chromosome 11. Many GWAS have found associations with SNPs in *DHCR7* and VD concentrations [49,50]. While earlier studies have reported that these SNPs are within *DHCR7*, they have been assigned to *NADSYN1* in newer genome assemblies. Both genes are within a tightly linked chromosome loci (11q13.4) and are therefore presented as the *NADSYN1/DHCR7* loci [71].

Additionally, several de novo mutations in *DHCR7* that lead to reduced DHCR7 expression during fetal development are responsible for Smith-Lemli-Opitz syndrome (SLOS), a rare inherited autosomal recessive disease [72]. It has been proposed that mutations in *DHCR7* may offer a health advantage at latitudes distal to the equator where exposure to intense UVB via sunlight at temperatures conducive to optimal thermal reactions in the skin are rare for much of the year [72]. In the context of VD synthesis, lower amounts of functional DHCR7 would reduce conversion of 7-DHC to cholesterol, thus increasing 7-DHC availability for VD synthesis in sunlight, which would help protect against diseases like rickets [72].

### 3.3. Cholesterol-Transport Proteins (CTPs)

Cholesterol-transport proteins (CTPs) facilitate the uptake of dietary VD and other fat-soluble vitamins across the intestinal epithelium, their transport from enterocytes into circulation, delivery to target tissues, and biliary excretion [73]. Key CTPs include SR-B1 (encoded by *SCARB1* on chromosome 12), *CD36* (on chromosome 7), and *NPC1L1* (also on chromosome 7). These proteins transport VD, carotenoids, α-tocopherol, vitamin K, and other lipophilic compounds throughout the body.

Evidence from knockout mouse models supports their potential role in regulating VD levels. *SCARB1*-deficient mice exhibited higher concentrations of deuterated cholecalciferol in serum, adipose tissue, kidney, and heart, but lower deuterated calcidiol in serum, liver, and kidney. *CD36*-deficient mice showed no difference in cholecalciferol compared to wild-type controls, but had reduced calcidiol in serum, liver, and kidney [74]. These findings suggest that both SR-B1 and CD36 may influence VD levels and tissue distribution.

Although direct associations between human CTP gene variants and serum calcidiol are lacking, established effects on the transport of other fat-soluble vitamins make their involvement biologically plausible. With regard to *SCARB1*, variants such as rs5888, rs11057830, and the missense mutation rs4238001 can affect protein function, with rs11057830 linked to lower plasma vitamin E [52]. Certain *SCARB1* haplotypes, CCT (rs5888, rs4238001, rs61932577) for example, were associated with lower plasma provitamin A carotenoids compared to the more common TCC haplotype [51]. For *CD36*, the haplotype GGACC (rs1984112, rs1761667, rs1527479, rs1527483, rs13230419) is linked to higher plasma carotenoids, and rs3211958 is associated with α-tocopherol concentrations in adipose tissue [51,53]. *NPC1L1* variants also show metabolic relevance: rs2072183 differs in genotype frequency between healthy and dyslipidemic Japanese subjects [54] and correlates with lipid profiles among Chinese ethnic groups [55]. Sequencing of *NPC1L1* in a Chinese population identified a promoter variant (g.-762T>C) that increased transcriptional activity and was associated with higher serum cholesterol [56]. Additional nonsynonymous mutations in *NPC1L1* (rs62001882, rs141973731, rs139659653, rs114375162) have been proposed to influence vitamin E bioavailability [52].

While these associations remain speculative for VD and are not yet clinically actionable, they provide a rationale for targeted studies to determine whether CTP polymorphisms contribute to interindividual differences in VD absorption and status.

### 3.4. GC

The *GC* gene, located on chromosome 4, encodes DBP, the primary transporter of VD metabolites in circulation. DBP is synthesized mainly by hepatocytes, and its expression increases in response to estrogenic compounds, glucocorticoids, and inflammatory cytokines [75]. Although not essential for the entry of VD metabolites into target cells according to the free hormone hypothesis, DBP serves as a critical reservoir that buffers fluctuations in VD supply and helps prevent deficiency when synthesis is suboptimal [31]. DBP also binds to renal megalin (LRP2) receptors, enabling the reabsorption and recycling of VD metabolites in the kidney [75]. Binding affinity differs among metabolites, with VD_3_ derivatives generally binding more strongly than VD_2_. Reported affinities for VD_3_ metabolites rank as calcidiol-lactones > 24,25-dihydroxycholecalciferol > calcitriol [75].

*GC* is highly polymorphic, with over 27,000 rs entries in the NCBI dbSNP database. Two missense SNPs, rs7041 and rs4588, define the common GC isoforms *GC1s*, *GC1f*, and *GC2*, which differ in DBP concentration and VD binding affinity [49,50]. The *GC1f* allele has been linked to lower serum calcidiol levels and a higher risk of hypovitaminosis D in Chinese infants [76], while individuals homozygous for *GC2* typically have 5–10% lower DBP concentrations than those with GC1 [75]. Although no *GC* polymorphism completely abolishes DBP binding, rare cases of complete *GC* deletion have been reported. In 2019, Henderson et al. [77] described a patient with a homozygous *GC* deletion, absent DBP in serum, and extremely low calcidiol and calcitriol levels, that did not respond to UVB phototherapy or supplementation. Despite this, the patient did not develop rickets but later presented with osteopenia and fragility fractures, suggesting that local paracrine or intracrine activation of VD may partially compensate for low circulating levels. These observations highlight how GC variation can affect laboratory measures of VD status, physiological outcomes, and the interpretation of deficiency risk.

### 3.5. Cytochrome P450 Genes

#### 3.5.1. Cyp2r1

The gene coding CYP2R1 is found on human chromosome 11. *Cyp2r1* is primarily expressed in the liver and is responsible for the 25-hydroxylation of cholecalciferol to calcidiol. Several CGAS and GWAS involving different ethnic groups have revealed associations between SNPs in *cyp2r1* (Table 1) and VD status [49,50,57,59]. A study by Casella et al. also interrogated 44 nonsynonymous polymorphisms in *cyp2r1* to determine if they influence calcidiol concentrations in blood [78]. These authors found that 21 of these polymorphisms decreased CYP2R1 activity, whereas 2 increased CYP2R1 activity. Interestingly, they also found that the frequency of polymorphisms decreasing CYP2R1 activity was greater in African-Americans than Caucasians, leading them to hypothesize that positive selection for non-functional alleles occurs closer to the equator, and this hypothesis was further supported by them correlating the frequency of inactivating *GC* variants with latitude.

Dong et al. recently reviewed the functional implications of several polymorphisms in *cyp2r1* [60]. The authors emphasized that 6359T>C SNP in exon 2 (designated CYP2R1*2) causes protein misfolding that may negatively impact CYP2R1 activity. They also reported on a SNP (5’UTR rs10741657) that may affect *cyp2r1* expression and activity. The rs10741657 risk allele has also been reported to be associated with a 3.7-fold higher likelihood of VD insufficiency in a small study involving 180 patients in the United States of America [61].

Although polymorphisms within *cyp2r1* account for some of the variation in calcidiol concentrations in blood, two missense mutations in *cyp2r1* (L99P and K242N) have also been found in Nigerian families having rickets [79]. The authors demonstrated that calcidiol production after an oral bolus dose of cholecalciferol (50,000 IU) was lower in heterozygous subjects and minimal in homozygous subjects compared to those not having these mutations. They also validated this work in vitro using HEK293 cells with the same mutations.

#### 3.5.2. Cyp27a1

*Cyp27a1* is located on human chromosome 2 and is mostly expressed in the liver. Like CYP2R1, it also contributes to the 25-hydroxylation of cholecalciferol to calcidiol. *Cyp27a1* is polymorphic, but *cyp27a1* SNPs (intronic rs17470271, intronic rs933994) were not found to be associated with a VD-associated disease (hepatitis) in a high-risk Chinese population, in contrast to *cyp2r1* SNPs (rs12794714, intronic rs10741657, intronic rs1562902, and rs10766197), which were [58]. Jarrar et al. proposed that CYP27A1’s role in the 25-hydroxylation of cholecalciferol is secondary to CYP2R1 [64]. However, it would be interesting to investigate if its contribution increases for individuals having attenuated CYP2R1 function; this was also suggested by Norlin & Wikvall [80].

#### 3.5.3. Cyp27b1

*Cyp27b1* is located on human chromosome 12 and is highly expressed in the kidney. CYP27B1 is responsible for the 1α-hydroxylation of calcidiol to bioactive calcitriol. As discussed earlier, Jolliffe et al. reviewed associations between SNPs in *cyp27b1* and circulating VD concentrations (Table 1) [49]. More recently, Hu et al. found that diabetic patients having the G/T genotype at rs10877012 were more frequently (71.79%) able to achieve calcidiol concentrations in blood > 30 ng/mL than other genotypes after VD supplementation, even though their basal levels before supplementation across genotypes were not different [62]. Another SNP (intronic rs4646536), has also been found to be strongly associated with risk of VD deficiency in an Iranian population [63]. Lastly, *cyp27b1* loss-of-function mutations are rare, but are the cause of the autosomal recessive disorder VD-dependent rickets type 1A (VDDR1A). Patients with VDDR1A having biallelic missense or frameshift mutations in *cyp27b1* have low to undetectable concentrations of calcitriol despite having normal calcidiol concentrations, and require life-long supplementation with calcitriol [81].

#### 3.5.4. Cyp24a1

*Cyp24a1* is located on human chromosome 20 and is highly expressed in the kidney. CYP24A1 is responsible for inactivating calcitriol via 24-hydroxylation. In the review by Jarrar et al., several SNPs (intronic rs2248137, rs2296241, intronic rs927650) in *cyp24a1* were reported to be associated with VD concentrations, and several other SNP associations have been reported elsewhere (Table 1) [64]. For example, a case–control study comprising 114 healthy controls and 239 VD-deficient subjects recently found the genotypes of SNPs (intronic rs4809960, intronic rs2585428) to be associated with risk of VD deficiency [65]. Becerra-Cervera et al. also recently reported associations with a SNP (rs6013897) and VD status [66]. Lai et al. previously reported associations with a SNP (rs172167070) and calcidiol concentrations and VD responder status in children with cystic fibrosis [67]. Gotoh-Saito et al. recently identified a *cyp24a1* enhancer (DEP128) containing two SNPs (rs6013892, rs158523) previously associated with VD concentrations [68]. The authors demonstrated by reported gene assay using ShP51 cells that haplotypes constructed from these SNPs had different gene regulatory activity. Lastly, a case study by Leszczyńska et al. identified two *cyp24a1* loss-of-function mutations (rs114368325, rs777676129) in a hyperthyroid patient with hypercalcemia. LC-MS/MS analysis of VD metabolites revealed high concentrations of cholecalciferol and low concentrations of a CYP24A1 VD metabolite (24,25-dihydroxycholecalciferol), suggesting a novel defect in VD catabolism [69].

#### 3.5.5. Cyp3a4

*Cyp3a4* is found on human chromosome 7. CYP3A4 is widely known for its role in metabolizing drugs, steroids, and xenobiotics. For example, tacrolimus, a calcineurin inhibitor used to prevent rejection of renal transplants, is metabolized by CYP3A4, and genetic variants in *cyp3a4* (cyp3a4*22, cyp3a4*18B) have been found to influence the pharmacokinetics of this immunosuppressive drug [82]. Since hepatic CYP3A4 also participates in the inactivation of both calcidiol and calcitriol, it is reasonable to hypothesize that genetic variants in *cyp3a4* will also affect VD concentrations. In support of this, a de novo *cyp3a4* missense mutation (c.902T>C) that resulted in a 10-fold increase in CYP3A4 activity was revealed in two patients with rickets that had low concentrations of calcidiol and calcitriol in their serum [70]. Follow-up reporter gene assay experiments by this group revealed that the outcome of isoleucine replacement by threonine at codon 301 led to gain-of-function of CYP3A4 that accelerated VD metabolism.

#### 3.5.6. Cyp11a1

*Cyp11a1* is found on human chromosome 15. *Cyp11a1* is most highly expressed in the skin, adrenal glands, testes, ovaries, and placenta, and is the first enzyme involved in steroidogenesis, converting cholesterol to pregnenolone. CYP11A1 can also hydroxylate 7-DHC, cholecalciferol, L3, and T3 into bioactive metabolites that can be detected in the circulation at comparable or greater concentrations than calcidiol [19]. Associations with polymorphisms or mutations in *cyp11a1* have been reported in the context of adrenal insufficiency and disorders of sex development [83,84], but we are unaware of any reported associations with VD concentrations. Yin et al. recently reported higher murine serum cholesterol, pregnenolone, and progesterone concentrations in offspring from placental trophoblast-specific *cyp11a1* Hipp11 (H11) knock-in dams [85]. Unexpectedly, these authors also reported lower serum calcidiol concentrations in these offspring, but not in the offspring of dams that had received supplementation with 5000 IU/kg of cholecalciferol during pregnancy [85]. The results of this study suggest that tissue-specific variants in *cyp11a1* have the potential to affect concentrations of circulating bioactive VD metabolites, but more research is needed to validate this and to better understand its potential implications.

## 4. Summary of Genetic Findings

Numerous genetic variants have been implicated in the regulation of VD synthesis, transport, and metabolism, with polymorphisms or mutations in genes such as *DHCR7/NADSYN1*, *GC*, *cyp2r1*, *cyp24a1*, and *cyp27b1* consistently associated with serum calcidiol concentrations across diverse populations and ethnicities. These genes encode proteins critical to various levels of VD regulation. For example, *DHCR7* regulates cutaneous synthesis by controlling the availability of 7-dehydrocholesterol, the precursor for VD3 production in the skin. Variants in *GC* affect the affinity and circulating levels of DBP, thereby influencing concentrations of calcidiol as well as other VD metabolites. CYP2R1 and CYP27A1 are involved in the hepatic 25-hydroxylation of cholecalciferol, with *cyp2r1* variants particularly linked to VD insufficiency and rickets in some populations. Genes such as *cyp27b1*, *cyp24a1*, and *cyp3a4* govern the activation of calcidiol to calcitriol and its subsequent inactivation, and their polymorphisms or mutations can significantly alter VD bioavailability and catabolism. Additionally, genes encoding intestinal cholesterol transporters—*SCARB1*, *CD36*, and *NPC1L1*—may influence the absorption of dietary VD, although these associations are less well established. Collectively, these findings support the view that VD status is a polygenic trait and suggest that the integration of genetic information in a clinical environment, such as through genomic risk scores (GRS), may enhance the precision of dietary recommendations and help optimize individual serum calcidiol levels.

## 5. Clinical Implications

Genetic variants can influence an individual’s ability to synthesize, transport, or metabolize VD. Although these insights hold promise for personalized supplementation strategies, routine genetic testing is not yet supported by large-scale clinical validation. For now, clinicians should rely on phenotype-based assessments to identify those at higher risk of VD deficiency, such as individuals with darker skin pigmentation, limited sun exposure, low dietary VD intake, or a family history of rickets, osteoporosis, or autoimmune disease. In such cases, standard supplementation protocols may be insufficient, and individualized dosing should be considered, especially for patients who do not achieve adequate serum calcidiol levels despite conventional mitigation approaches.

A high-dose cholecalciferol challenge, as demonstrated by O’Hearn et al. and Helmeczi et al. [86,87], could help evaluate VD bioavailability, half-life, and metabolite patterns in individuals with known genotypes, or suspected metabolic impairments. In these studies, a single oral dose of 10,000 IU/kg (maximum 400,000 IU) produced a rapid increase in serum calcidiol levels without adverse effects, followed by a gradual decline to baseline over approximately one month. For patients with persistently low VD status, despite adequate intake and no clear environmental or dietary cause, alternative formulations, such as calcidiol or calcitriol, may be more appropriate to bypass early metabolic steps affected by certain genetic variants.

Individuals with a family history of unexplained VD-related disorders or with poor responses to high-dose supplementation may also benefit from referral for genetic counseling. These approaches remain exploratory and should be applied cautiously, ideally within research settings, or when conventional strategies have failed. As the evidence base grows, integration of validated genetic data into clinical decision-making may help to refine supplementation guidelines to improve outcomes. In the meantime, a phenotype-driven strategy that accounts for possible genetic variability offers a practical path toward more tailored VD management.

## 6. Concluding Remarks

Vitamin D status is a polygenic trait influenced by polymorphisms in genes regulating VD synthesis, transport, and metabolism, as well as by rare de novo mutations that can markedly alter VD metabolism. These genetic factors, together with seasonal variation, mean that adequate serum levels cannot always be achieved through standard dietary recommendations alone. Variants within the VDR may also indirectly influence circulating metabolite levels by altering the regulation of genes, such as CYP24A1, that contain VDREs. Such genetic diversity, which varies across populations of different ancestries, underscores the importance of regular assessment of VD status and consideration of individual genetic risk for deficiency or excess.

Understanding the combined effects of genetics, environment, and gene-by-environment interactions is essential for developing precise dietary recommendations. Genomic risk scores (GRSs) have been proposed as tools to predict VD deficiency, toxicity risk, and related health outcomes, and could one day help tailor supplementation. However, current GRS models remain research tools, often derived from European ancestry populations, and require validation in large, ethnically diverse cohorts. These models must also integrate non-genetic factors such as sun exposure, latitude, diet, and skin pigmentation. Once validated, GRS approaches could help predict the intake required for optimal VD levels and inform precision nutrition strategies.

## Figures and Tables

**Table 1:** Summary of reference sequences (rs), reference sources, and NCBI functional consequences of variants in genes with the potential to regulate vitamin D synthesis, transport, and metabolism.

	Gene	NCBI Functional Consequence	Functional Role	Reference
rs12785878	*DHCR7/NADSYN1*	Intronic	Regulates precursor availability (7-DHC)	[49]
rs3829251	*DHCR7/NADSYN1*	Intronic	Regulates precursor availability (7-DHC)	[49]
rs7944926	*DHCR7/NADSYN1*	Intronic	Regulates precursor availability (7-DHC)	[49]
rs12800438	*DHCR7/NADSYN1*	Intronic	Regulates precursor availability (7-DHC)	[49]
rs3794060	*DHCR7/NADSYN1*	Intronic	Regulates precursor availability (7-DHC)	[49]
rs4944957	*DHCR7/NADSYN1*	Intronic	Regulates precursor availability (7-DHC)	[49]
rs4945008	*DHCR7/NADSYN1*	Unknown	Regulates precursor availability (7-DHC)	[49]
rs5888	*SCARB1*	Synonymous	Cholesterol/VD absorption and transport	[51,52]
rs11057830	*SCARB1*	Intronic	Cholesterol/VD absorption and transport	[52]
rs4238001	*SCARB1*	Missense	Cholesterol/VD absorption and transport	[51,52]
rs61932577	*SCARB1*	Intronic	Cholesterol/VD absorption and transport	[51]
rs1984112	*CD36*	Intronic	Cholesterol/VD absorption and transport	[51]
rs1761667	*CD36*	Intronic	Cholesterol/VD absorption and transport	[51]
rs1527479	*CD36*	Intronic	Cholesterol/VD absorption and transport	[51]
rs1527483	*CD36*	Intronic	Cholesterol/VD absorption and transport	[51]
rs13230419	*CD36*	Unknown	Cholesterol/VD absorption and transport	[51]
rs3211958	*CD36*	Intronic	Cholesterol/VD absorption and transport	[53]
rs2072183	*NPC1L1*	Synonymous	Cholesterol/VD absorption and transport	[54,55]
rs62001882	*NPC1L1*	Missense	Cholesterol/VD absorption and transport	[52]
rs141973731	*NPC1L1*	Missense	Cholesterol/VD absorption and transport	[52]
rs139659653	*NPC1L1*	Missense	Cholesterol/VD absorption and transport	[52]
rs114375162	*NPC1L1*	Stop gained	Cholesterol/VD absorption and transport	[52]
g.-762T>C	*NPC1L1*	Unknown	Cholesterol/VD absorption and transport	[56]
rs4588	*GC*	Missense	Affects VD binding protein affinity and levels	[49]
rs7041	*GC*	Missense	Affects VD binding protein affinity and levels	[49]
rs1155563	*GC*	Intronic	Affects VD binding protein affinity and levels	[49]
rs17467825	*GC*	Unknown	Affects VD binding protein affinity and levels	[49]
rs2070741	*GC*	Intronic	Affects VD binding protein affinity and levels	[49]
rs2298849	*GC*	Intronic	Affects VD binding protein affinity and levels	[49]
rs16846876	*GC*	Unknown	Affects VD binding protein affinity and levels	[49]
rs842999	*GC*	Intronic	Affects VD binding protein affinity and levels	[49]
rs222035	*GC*	Intronic	Affects VD binding protein affinity and levels	[49]
rs3755967	*GC*	Intronic	Affects VD binding protein affinity and levels	[49]
rs2298850	*GC*	Intronic	Affects VD binding protein affinity and levels	[49]
rs12512631	*GC*	Unknown	Affects VD binding protein affinity and levels	[49]
rs2282679	*GC*	Intronic	Affects VD binding protein affinity and levels	[49]
rs705117	*GC*	Intronic	Affects VD binding protein affinity and levels	[50]
rs1352846	*GC*	Intronic	Affects VD binding protein affinity and levels	[50]
rs10741657	*cyp2r1*	2KB Upstream	Catalyzes 25-hydroxylation	[49,57]
rs2060793	*cyp2r1*	2KB Upstream	Catalyzes 25-hydroxylation	[49]
rs1993116	*cyp2r1*	Intronic	Catalyzes 25-hydroxylation	[49,57]
rs7116978	*cyp2r1*	Intronic	Catalyzes 25-hydroxylation	[49,57]
rs12794714	*cyp2r1*	Synonymous	Catalyzes 25-hydroxylation	[49,50,57,58]
rs10500804	*cyp2r1*	Intronic	Catalyzes 25-hydroxylation	[49,57]
rs10766197	*cyp2r1*	Unknown	Catalyzes 25-hydroxylation	[49,58]
rs2060793	*cyp2r1*	2KB Upstream	Catalyzes 25-hydroxylation	[59]
rs10741657	*cyp2r1*	5’UTR	Catalyzes 25-hydroxylation	[58,60,61]
rs1562902	*cyp2r1*	Unknown	Catalyzes 25-hydroxylation	[58]
6359T>C	*cyp2r1*	Unknown	Catalyzes 25-hydroxylation	[60]
rs17470271	*cyp27a1*	Intronic	Catalyzes 25-hydroxylation	[58]
rs933994	*cyp27a1*	Intronic	Catalyzes 25-hydroxylation	[58]
rs10877012	*cyp27b1*	2KB Upstream	Converts 25(OH)D to active form (calcitriol)	[49,62]
rs118204009	*cyp27b1*	Missense	Converts 25(OH)D to active form (calcitriol)	[49]
rs4646536	*cyp27b1*	Intronic	Converts 25(OH)D to active form (calcitriol)	[49,63]
rs2248137	*cyp24a1*	Intronic	Inactivates calcitriol via hydroxylation	[64]
rs2296241	*cyp24a1*	Synonymous	Inactivates calcitriol via hydroxylation	[64]
rs927650	*cyp24a1*	Intronic	Inactivates calcitriol via hydroxylation	[64]
rs4809960	*cyp24a1*	Intronic	Inactivates calcitriol via hydroxylation	[65]
rs2585428	*cyp24a1*	Intronic	Inactivates calcitriol via hydroxylation	[65]
rs6013897	*cyp24a1*	Unknown	Inactivates calcitriol via hydroxylation	[66]
rs17216707	*cyp24a1*	Unknown	Inactivates calcitriol via hydroxylation	[67]
rs6013892	*cyp24a1*	Unknown	Inactivates calcitriol via hydroxylation	[68]
rs158523	*cyp24a1*	Unknown	Inactivates calcitriol via hydroxylation	[68]
rs114368325	*cyp24a1*	Missense	Inactivates calcitriol via hydroxylation	[69]
rs777676129	*cyp24a1*	Inframe Deletion	Inactivates calcitriol via hydroxylation	[69]
c.902T>C	*cyp3a4*	Unknown	Alternative inactivation pathway (hepatic)	[70]

## Abbreviations

Candidate gene association study (CGAS), cholecalciferol (VD_3_), cholesterol-transport proteins (CTPs), cytochrome P450 (CYP), 7-dehydrocholesterol (7-DHC), 7-dehydrocholesterol reductase (DHCR7), genome wide association study (GWAS), genomic risk score (GRS), knockout (KO), lumisterol (L3), Niemann-Pick C1-Like 1 (NPC1L1), parathyroid hormone (PTH), pregnane X receptor (PXR), renal megalin membrane receptor (LRP2), scavenger receptor class B type 1 (SR-B1), single nucleotide polymorphisms (SNPs), Smith-Lemli-Opitz syndrome (SLOS), tachysterol (T3), ultraviolet B (UVB), vitamin D (VD), vitamin D binding protein (DBP), VD receptor (VDR), VD response elements (VDREs).
